# In Vitro Comparison of Various High-Speed Vitrectomy Machines Using Dual Blade Cutters

**DOI:** 10.7759/cureus.15021

**Published:** 2021-05-14

**Authors:** Haroon Tayyab, saima jamil, Shiraz Hashmi

**Affiliations:** 1 Surgery/Ophthalmology, The Aga Khan University Hospital, Karachi, PAK; 2 Surgery, The Aga Khan University Hospital, Karachi, PAK

**Keywords:** vitrectomy, vitreous, pars plana, duty cycle, infusion pressure

## Abstract

Objectives

The objective of this study is to compare various dual blade vitrectomy cutters for their efficiency in an in vitro setting.

Methods

In this in vitro experimental study, we compared various vitrectomy systems including EVA (Dutch Ophthalmic Research Center, Zuidland, The Netherlands), REVOLUTION (Optikon 2000, Inc., Rome, Italy), and OS4 (Oertli Instrumente AG, Berneck, Switzerland) in terms of efficiency in vitreous cutting and aspiration for various vitreous substitutes. These substitutes included water, chicken egg albumin, and goat vitreous. We only used 23-gauge dual blade cutters across all platforms to maintain uniformity. The cutting and aspiration efficiency was measured across various cut and vacuum settings of vitrectomy machines and measured as mass aspirated in a given time. Data analysis included comparing the amount of mass aspirated by these machines at preset cut and vacuum settings.

Results

Scatter plots showed a comparable mass of water aspirated by the EVA and REVOLUTION at 1000 to 5000 cuts per minute at a constant vacuum of 500 mm Hg whereas OS4 aspirated lesser mass at similar settings. Same trends were noted for goat vitreous for EVA and REVOLUTION but aspirated mass of albumin fluctuated widely across various platforms. At peak machine settings, REVOLUTION showed superiority across all three vitreous models due to its higher peak settings. The area under curve (AUC) analysis showed no significant differences among machines for water and goat vitreous at comparable settings but results were fluctuating for egg albumin.

Conclusion

Employing higher cut rates for dual blade cutters results in better efficiency of vitrectomy machines.

## Introduction

Ophthalmologists have been striving for new ideas of vitreous removal through a small opening without incurring much trauma to surrounding structures for more than four decades now. The initial humble beginnings were by Robert Machemer in 1971 when he introduced a vitreous cutter driven through a small electric motor mounted on a syringe with a needle [1]. This multifunction (cut, infusion, aspiration) vitreous cutter was 17-gauge with a diameter of 1.42 mm and utilized a 2.3 mm scleral opening [2]. Soon after the inception of single port multipurpose vitrectomy, O’Malley introduced a three-port pars plana vitrectomy utilizing a 20-gauge guillotine cutter and instruments. With growing interest and ability to manage more complex retinal disorders, not only the gauge of instruments became smaller, but the cutting and fluidics also improved significantly. In 2002, Fujii et al. introduced 25-gauge transconjunctival high-speed vitrectomy cutters which popularized the idea of small gauge vitrectomy [3]. Later on, Eckardt designed and introduced a 23-gauge transconjunctival vitrectomy. These instruments were more rigid in nature as opposed to 25-gauge instruments [4]. Currently, vitreoretinal surgeons use all these gauges across the globe with more interest in 27-gauge cutters and instruments introduced by Blinder et al. [5]. Despite progressive optimization of guillotine-based cutters that has resulted in higher cut rates, smaller gauge, less vitreous turbulence, and better fluidics, recently, a hypersonic vitrectomy cutter (Vitesse Hypersonic Vitrectomy: Bausch + Lomb, NJ, USA) has been approved for use by Food and Drug Administration. The mechanism of action is through vitreous liquefaction rather than vitreous cutting, thereby eliminating potential vitreous turbulence and retinal traction. But this technique is not in widespread use at the moment [6].

With understandable limitations on the minimalistic size and designs of vitreous cutters, more recent interest has been in the development of dual blade/twin-blade cutters that effectively double the cut-rate; and at the same time, provide more stable and efficient vitreous fluidics. These dual blade cutters can work very close to the retinal surface and because of their better fluidics, provide more traction-free vitreous cutting. These cutters are also based on the same guillotine cutting mechanism. A notable example has been of HYPERVITâ Dual Blade Vitrectomy Probe (Constellationâ Vison System, Alcon, Fort Worth, Texas). This allows an effective cut rate of up to 20,000 cuts per minute (CPM) with improved fluidics [7]. Furthermore, Dutch Ophthalmic Research Center (DORC) has recently introduced a two-dimensional cutting (TDC) vitrectome with twin-angled blades that promise cut rates up to 16,000 CPM and vastly improved fluidics [8]. Other similar novel twin blade cutter designs include constant flow blade (CFB; Twedge Cutter Blade; Optikon 2000, Inc., Rome, Italy) offering up to 20,000 CPM, Mach2 in megaTRON S4HSP (Geuder, Heidelberg, Germany) and Continuous Flow Cutter in OS4 (Oertli Instrumente AG, Berneck, Switzerland) offering 10,000 CPM [9].

The faster cut rates and small gauge vitrectors have also paved the way for higher infusion pressures. These infusion pressures range from 30 mmHg for 20-gauge vitrectomy up to 50 mmHg for 25- and 27-gauge vitrectomy systems. This arrangement facilitates better flow rates through small-bore instruments.

The next parameter governing the overall fluidics of the vitrectomy machine is aspiration flow rate. The aspiration flow rate is dependent on the preset vacuum and viscosity of the aspirated medium. In real-life scenarios, the aspiration flow rate is difficult to predict due to alternating viscosity encountered when aspirating vitreous, exudates, blood, and balanced salt solution at the same or subsequent phases of vitrectomy. Aspiration flow rate and consequent fluid accelerations are directly linked with stresses on the retinal surface and the overall efficiency of vitreous removal. To achieve the optimum balance between safety and efficacy, we may require the highest flow rate with the lowest vitreous accelerations around the cutter tip. This is principally controlled by the type of pump (venturi, peristaltic) being used but is also controlled by the duty cycle. Single blade cutters tend to have a lower duty cycle at higher cut speeds and thus more acceleration at the cutter tips. This factor can be mitigated by twin-blade cutters which achieve an open port state of cutter throughout the cut cycle [10-12].

The primary objective of this experimental study is to evaluate the efficiency of various vitrectomy platforms as a function of cutter speeds and vacuum in varying in vitro environments. To the best of our knowledge, no such comparison has been done before where these vitrectomy systems have been compared while using their respective dual blade cutters. This study will help in understanding the roles of higher cut rates with dual blade cutter and their ability to achieve more efficient vitreous removal against varying levels of vacuum generated by the machines. This will also allow retinal surgeons in third-world countries to make evidence-based decisions when procuring these very expensive machines in context with the overall efficiency of the machine.

## Materials and methods

Vitrectomy systems

In this in vitro experimental study, we used EVAä (Dutch Ophthalmic Research Center, Zuidland, The Netherlands), REVOLUTIONä (Optikon 2000, Inc., Rome, Italy), and OS4ä (Oertli Instrumente AG, Berneck, Switzerland) as vitrectomy platforms. EVAä vitrectomy system employs a peristaltic pump whereas REVOLUTIONä and OS4ä house both peristaltic and venturi pumps. We employed 23-gauge vitrectomy cutters for simplicity and comparison on a standardized approach, although smaller gauge cutters were available. For all experiments, we used the dual blade proprietary cutters respective for the machines. The specifics of individual machines are described in Table 1.

**Table 1:** Vitrectomy machine parameters for EVA, REVOLUTION, and OS4

Characteristics	EVA	REVOLUTION	OS4
Maximum CPM	8000 × 2	10,000 × 2	5000 × 2
Maximum aspiration rate (mmHg)	680	700	500
Type of pump	Peristaltic	Peristaltic + venturi	Peristaltic + venturi
Type of cutter	Two-dimensional cutting	Continuous flow cutter, Twedge	Continuous flow cutter

Vitreous models

We used goat eye vitreous, albumin (egg white), and distilled water as vitreous substitutes. The eggs were stored in a commercial refrigerator with a temperature display at 4 °C and all eggs were within their shelf-life. Goat eyes were obtained from a slaughterhouse and stored in normal saline before use. All goat eyes were used within eight hours of slaughter. No animal was purposefully harmed to obtain the vitreous samples. All experiments were conducted at 25 °C temperature.

Vitrectomy

All vitreous substitute samples were collected using 23-gauge dual blade cutters of respective vitrectomy platforms. The samples included goat eye vitreous, egg white albumin, and distilled water contained in a 40 ml borosilicate glass beaker. The samples were obtained at room temperature of 25 °C. The samples contained in the beaker were placed on a standard precise (up to 0.01 g) microbalance (Ohaus Corporation, Pine Brook, NJ, USA). Vitrectomy was initiated at 1000 CPM at 100 mmHg vacuum pressure. The cut-rate was incrementally increased by 1000 CPM till the maximum capacity of the respective machine was reached. Similarly, all cut-rate increments were measured against varying aspiration rates (increments of 100 mmHg) till the maximum aspiration rate of the respective machine was reached. The vitrectomy cutter was switched on for 30 seconds and three separate readings (mass in grams) were taken for every single setting. The average of the three readings (mass in grams) was considered as final for data analysis. The 30-second duration was recorded using a precise (up to 0.01 seconds) electronic stopwatch (Apple iPhone 11 pro Stopwatch, Apple, Inc., CA, USA). Measurement of aspirated mass in grams was recorded before the start of vitrectomy and then remeasured after 30 seconds of vitrectomy after removal of vitrector from the beaker.

Data analysis

Data were entered in an excel sheet and analyzed in MedCalc statistical software version 19.1.6 (MedCalc, Ostend, Belgium). For comparison of the efficiency of various vitrectomy systems in the study, graphs were built to visualize maximized aspirated mass (g) at various cut rates ranging from 0 to 10,000 CPM at a vacuum pressure of 100-700 (mmHg). Scatter plots presented for aspirated mass in grams at 500 mmHg vacuum pressure against the function of cut rates using the uppermost limit as 5000 CPM to make a uniform comparison for all three systems. Mean mass was computed for each machine for water, egg white, and goat vitreous. One-way Anova followed by Tukey HSD test to observe pairwise mean differences between the machine and by vitreous surrogates. A P-value of <0.05 was considered significant. The efficiency of machines was validated by computing comparative area under curves (AUCs) on the aspirated mass by each machine for water, egg white, and goat vitreous against the cut rates of 1000 and 4000 CPM, respectively. The difference in aspirated mass was estimated on the higher vacuum and cut rates.

## Results

**Figure 1:**
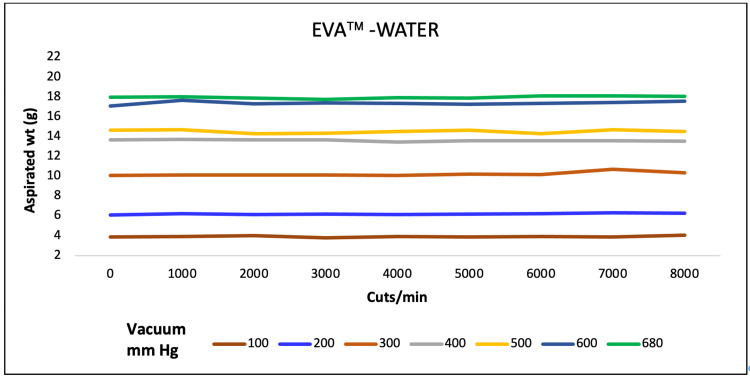
Aspiration of water (g) as a function of cut-rate and vacuum pressure for the EVA vitrectomy system

**Figure 2:**
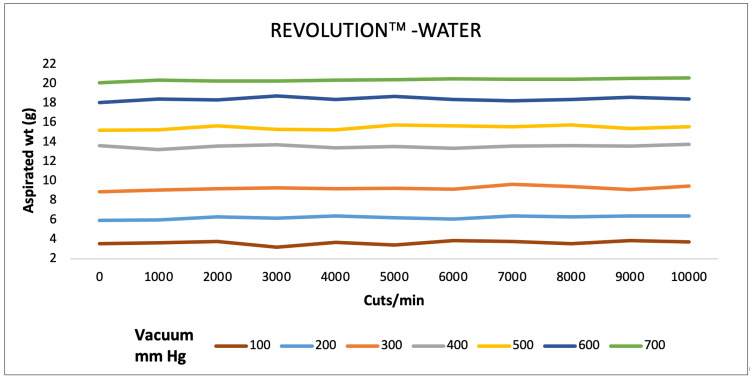
Aspiration of water (g) as a function of cut-rate and vacuum pressure for the REVOLUTION vitrectomy system

**Figure 3:**
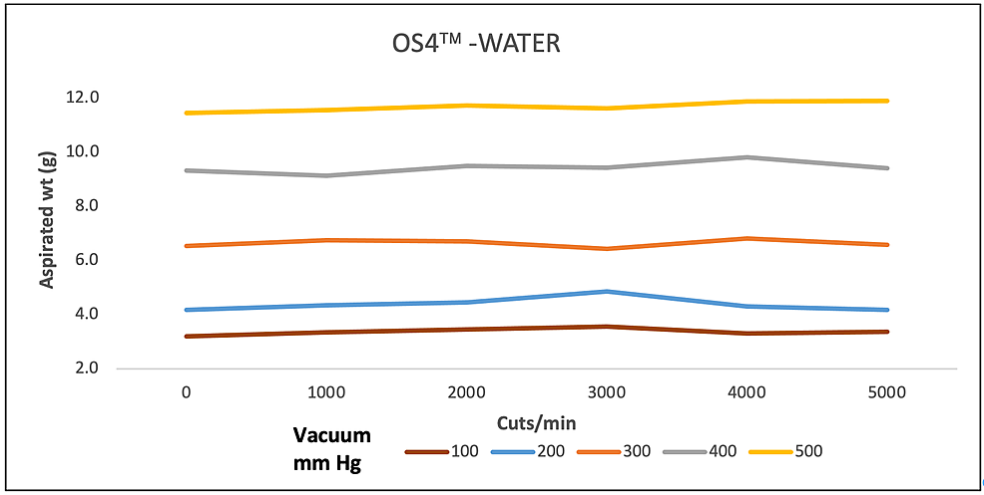
Aspiration of water (g) as a function of cut-rate and vacuum pressure for the OS4 vitrectomy system

A direct association of aspirated mass and CPM ranging from 1000 to 10,000 was observed at vacuum pressure varying from 100 to 700 mmHg for all three machines for water. EVA and REVOLUTION aspirated almost similar mass whereas OS4 aspirated low water mass (Figures 1-3).

**Figure 4:**
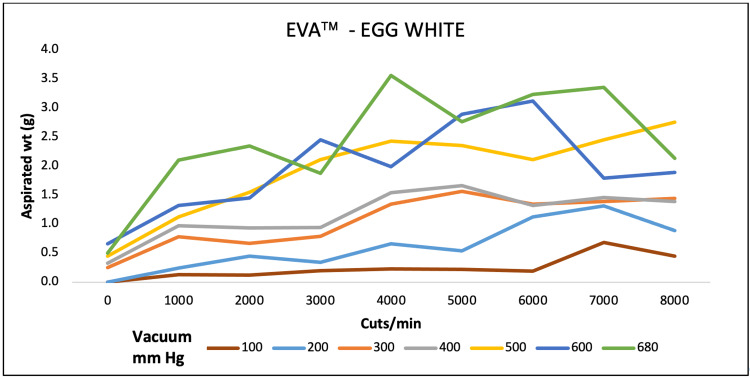
Aspiration of egg white (g) as a function of cut-rate and vacuum pressure for the EVA vitrectomy system

**Figure 5:**
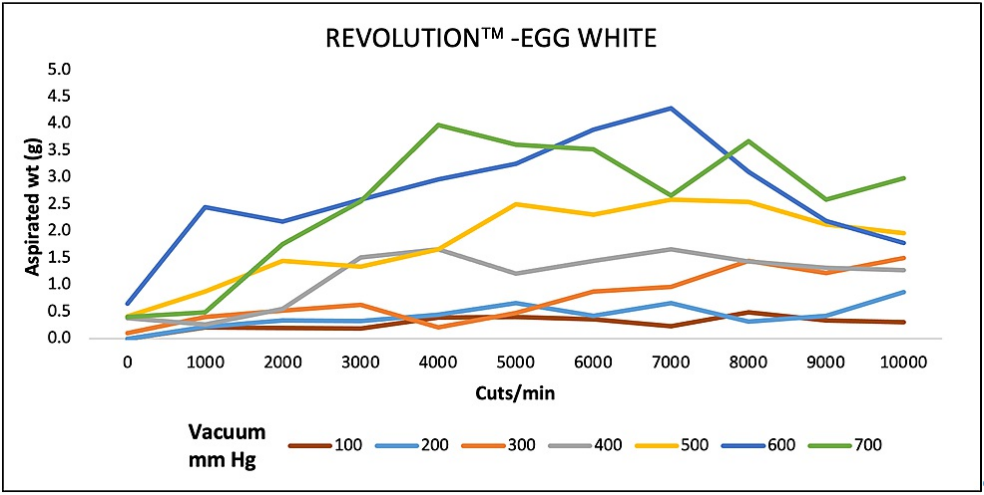
Aspiration of egg white (g) as a function of cut-rate and vacuum pressure for the REVOLUTION vitrectomy system

**Figure 6:**
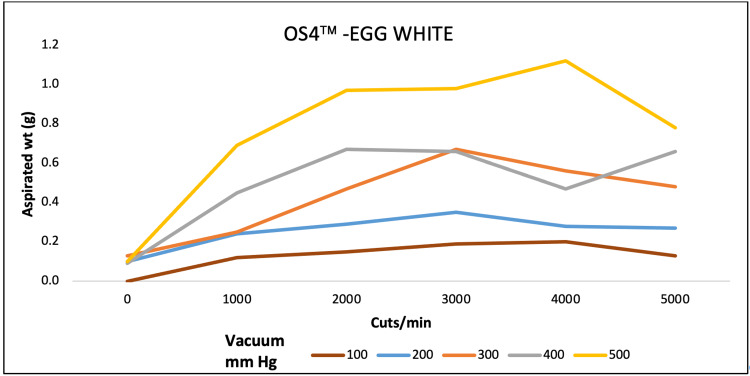
Aspiration of egg white (g) as a function of cut-rate and vacuum pressure for the OS4 vitrectomy system

For egg white, aspirated mass was higher at 4000 CPM both for EVA and OS4 at 680 and 500 mmHg of maximum pressure. For REVOLUTION, maximum aspiration was achieved at 7000 CPM and 4000 CPM at 600 and 700 mmHg pressure, respectively (Figures 4-6).

**Figure 7:**
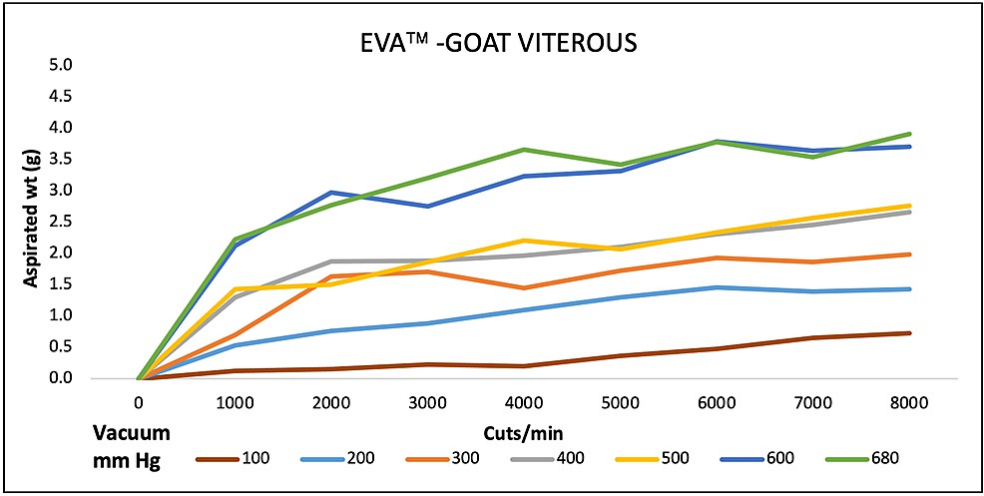
Aspiration of goat vitreous (g) as a function of cut-rate and vacuum pressure for the EVA vitrectomy system

**Figure 8:**
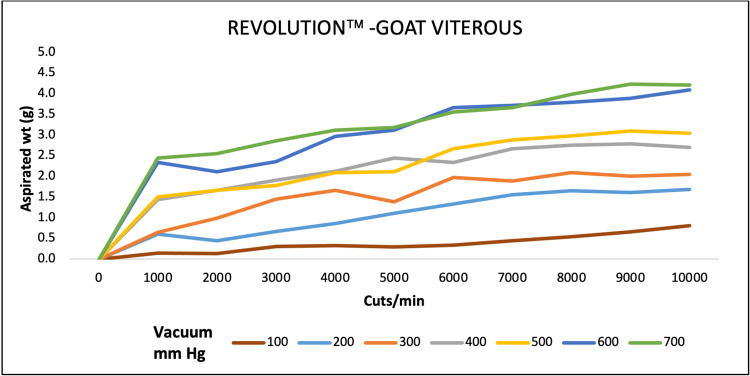
Aspiration of goat vitreous (g) as a function of cut-rate and vacuum pressure for the REVOLUTION vitrectomy system

**Figure 9:**
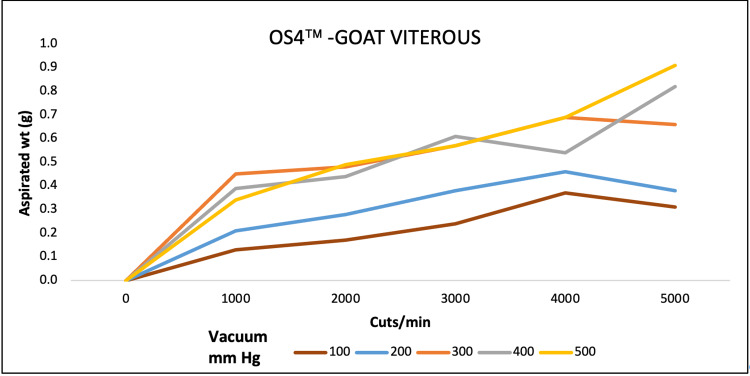
Aspiration of goat vitreous (g) as a function of cut-rate and vacuum pressure for the OS4 vitrectomy system

For goat vitreous aspiration, EVAä aspirated maximum at 4000, 6000, and 8000 CPM at 680 mmHg. REVOLUTIONä aspiration was maximum at 9000 CPM at a maximum pressure of 700 mmHg. OS4ä performance was high at maximum CPM and vacuum pressure of 5000 and 500, respectively (Figures 7-9).

Scatter plots

**Figure 10:**
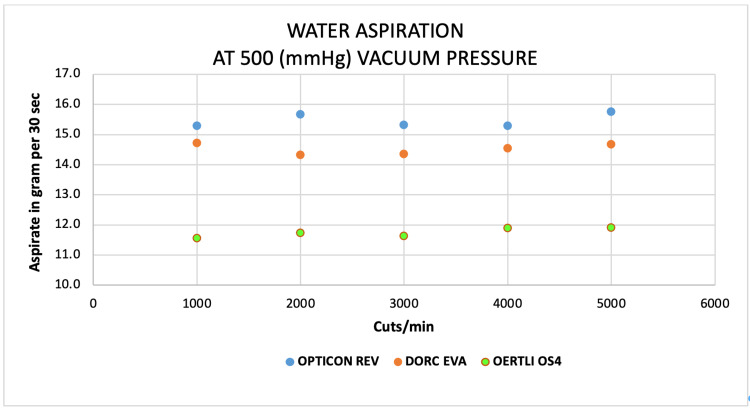
Scatter plots analysis of the aspiration of water (g) as a function of cut-rate and vacuum pressure at 500 mmHg for the EVA®, REVOLUTION®, and OS4® vitrectomy systems

**Figure 11:**
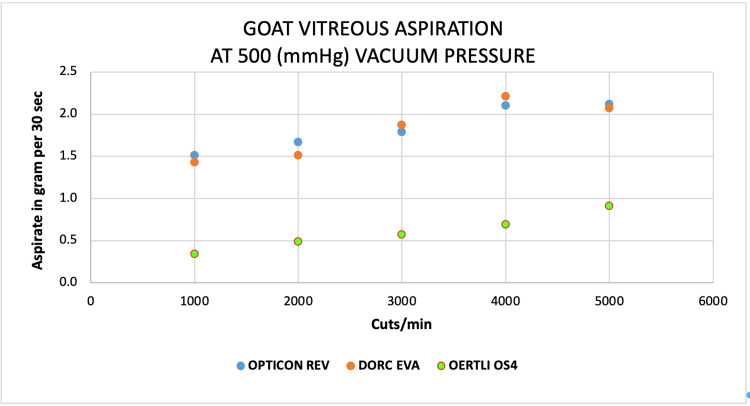
Scatter plots analysis of the aspiration of goat vitreous (g) as a function of cut-rate and vacuum pressure at 500 mmHg for the EVA®, REVOLUTION®, and OS4® vitrectomy systems

**Figure 12:**
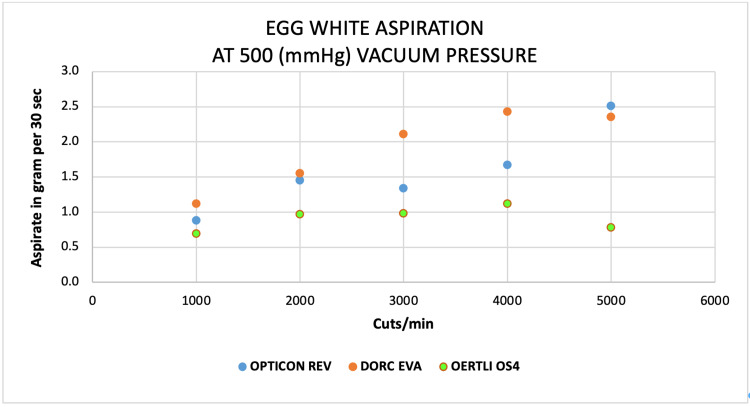
Scatter plots analysis of the aspiration of egg albumin (g) as a function of cut-rate and vacuum pressure at 500 mmHg for the EVA®, REVOLUTION®, and OS4® vitrectomy systems

Scatter plots show an almost comparable aspirated mass of water by both EVA and REVOLUTION at 1000 to 5000 CPM at a constant vacuum of 500 mmHg compared to a very low mass of water by OS4 system at the same parameters (Figure 10). Similar trends were found for goat vitreous aspiration on both EVA and REVOLUTION systems on the same thresholds (Figure 11). For egg-white albumin, the aspiration rates at 1000 and 5000 CPM were similar for both EVA and REVOLUTION systems; however, varied at 2000 to 4000 CPM on the same vacuum (Figure 12).

At 1000 cut rates, water aspiration was higher on REVOLUTION (12.3 ± 6.3 g) and low by OS4 (7.0 ± 3.4), whereas egg white aspiration was higher on EVA (0.95 ± 0.7) and low on OS4 (0.35 ± 0.2), for goat vitreous high aspirated mass observed on REVOLUTION and low by OS4 (1.3 ± 0.9 and 0.3 ± 0.1, respectively; Table 2). At 4000 cut rates, an almost similar pattern of aspiration was observed for every three surrogates (Table 3).

**Table 2:** Mean mass aspiration of vitreous surrogates by different systems at 1000 cut rates

Medium	Vitrectomy system	Mean	(±SD)
Water	EVA	12.1	5.5
	REVOLUTION	12.3	6.3
	OS4	7.0	3.4
Egg white albumin	EVA	0.95	0.7
	REVOLUTION	0.70	0.8
	OS4	0.35	0.2
Goat vitreous	EVA	1.2	0.8
	REVOLUTION	1.3	0.9
	OS4	0.3	0.1

**Table 3:** Mean mass aspiration of vitreous surrogates by different systems at 4000 cut rates

Medium	Vitrectomy system	Mean	(±SD)
Water	EVA	11.9	5.4
	REVOLUTIONä	12.4	6.2
	OS4	7.2	3.6
Egg white albumin	EVA	1.7	1.1
	REVOLUTION	1.6	1.4
	OS4ä	0.5	0.4
Goat vitreous	EVA	2.0	1.2
	REVOLUTION	1.9	1.0
	OS4	0.6	0.1

Mean mass difference at 1000 and 4000 cut rates for each of three surrogates were not statistically different (p>0.05); however, for goat vitreous aspiration, a higher trend could be seen for EVA compared to OS4 (p=0.057) and REVOLUTION compared to OS4 (p=0.078), but this high mass difference did not attain statistical significance. At the highest threshold cut rates of each machine (i.e., 5000, 8000, and 10,000) and vacuum pressure (i.e., 500, 680, and 700) REVOLUTION seems to be more efficient (Table 4).

**Table 4:** Aspirated mass and difference of mass at highest cut rates and vacuum for three vitreous substitutes

Medium	Vitrectomy system	Vacuum (mm Hg)	Cut-rate	Aspirated mass (g)	System difference	Difference of mass (g)
Water	EVA	680	8,000	18.07	REV-EVA	2.55
	REVOLUTION	700	10,000	20.62	REV-OS4	8.71
	OS4	500	5,000	11.91	EVA-OS4	6.16
Egg white albumin	EVA	680	8,000	2.13	REV-EVA	0.86
	REVOLUTION	700	10,000	2.99	REV-OS4	2.21
	OS4	500	5,000	0.78	EVA-OS4	1.35
Goat vitreous	EVA	680	8,000	3.91	REV-EVA	0.3
	REVOLUTION	700	10,000	4.21	REV-OS4	3.3
	OS4	500	5,000	0.91	EVA-OS4	3.0

**Figure 13:**
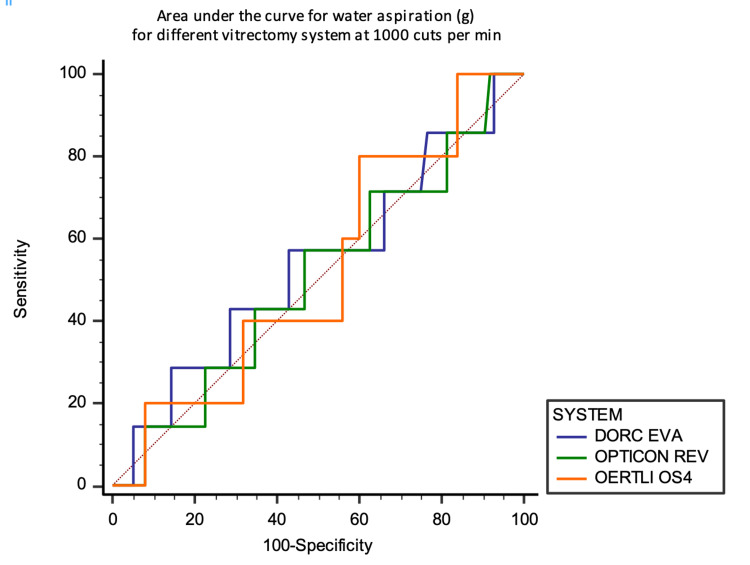
Area under the curve analysis at 1000 CPM against the aspirated mass of water in grams

**Figure 14:**
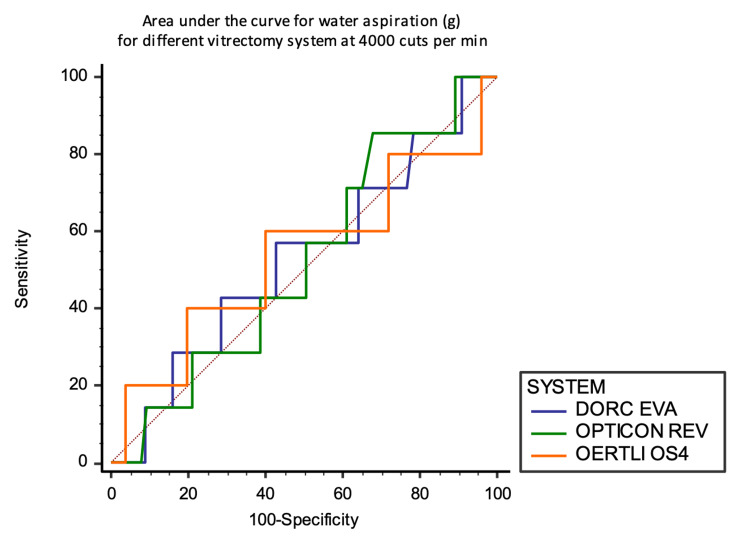
Area under the curve analysis at 4000 CPM against the aspirated mass of water in grams

Overall AUC analyses show no significant differences either at 1000 and 4000 CPM between the three systems at 1000 and 4000 mm Hg for water aspiration. AUCs fall around the diagonal line (0.5) (Figures 13 and 14).

**Figure 15:**
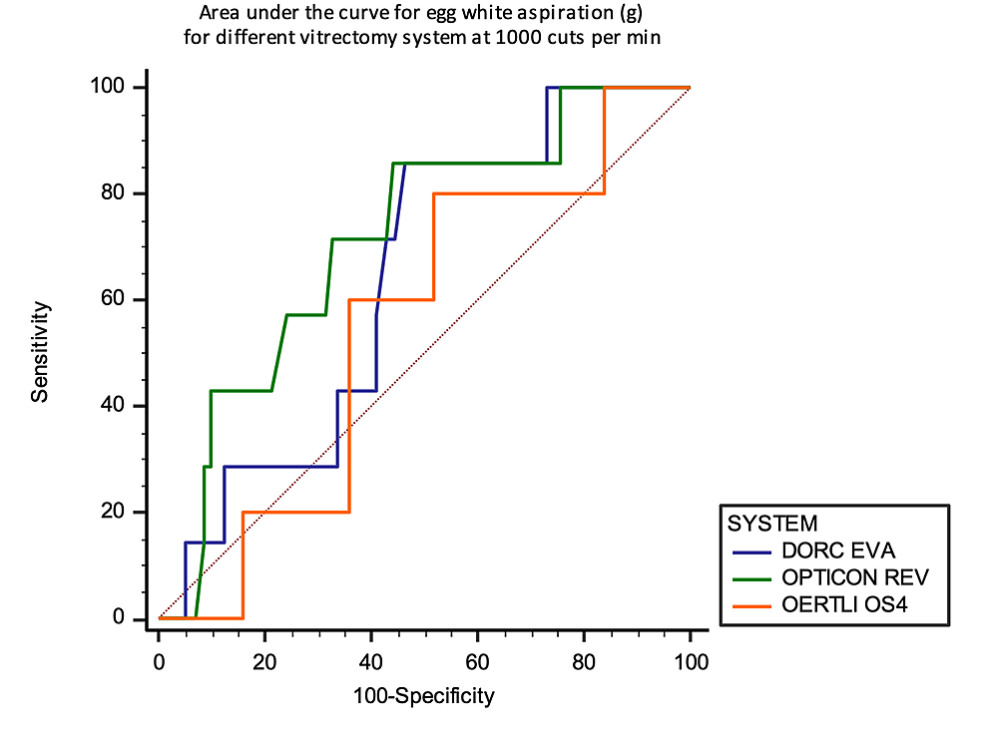
Area under the curve analysis at 1000 CPM against the aspirated mass of egg white in grams

**Figure 16:**
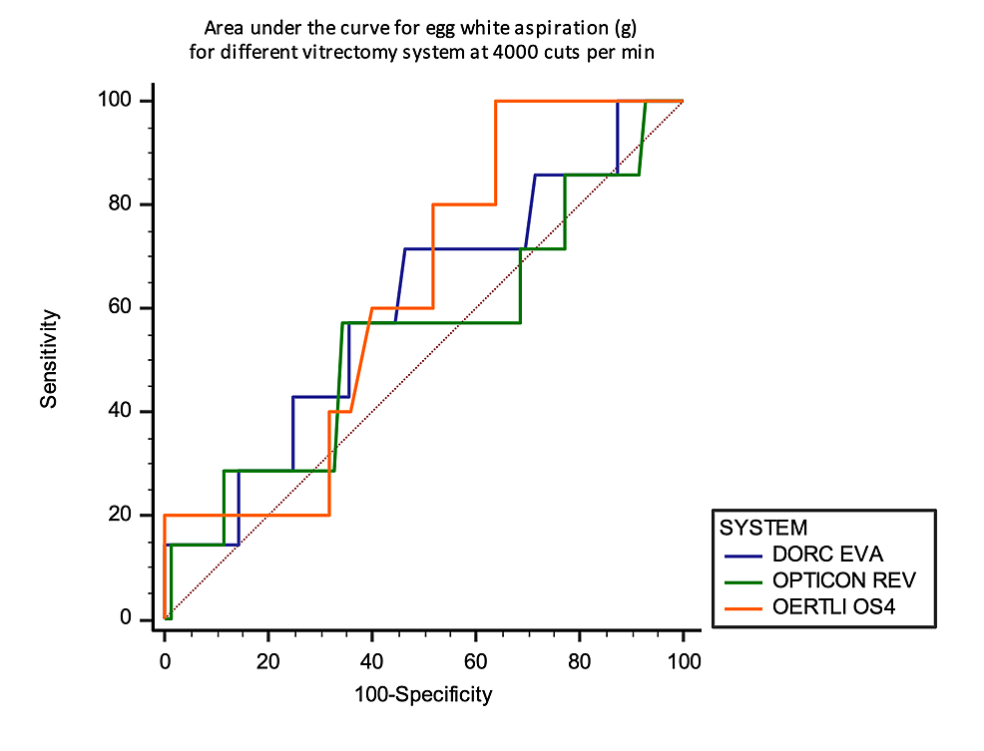
Area under the curve analysis at 4000 CPM against the aspirated mass of egg white in grams

However, for egg white aspiration, REVOLUTION showed a higher AUC of 0.713, 95% CI (0.518 to 0.909) at 1000 CPM compared to the other two machines at the same thresholds (Figures 15 and 16).

**Figure 17:**
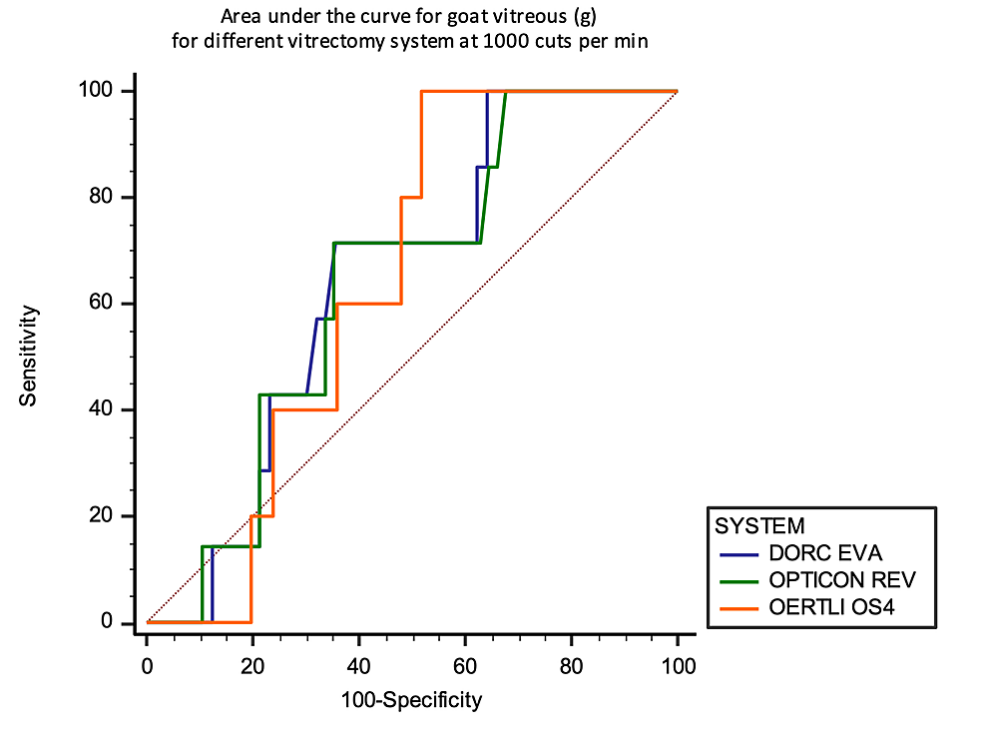
Area under the curve analysis at 1000 CPM against the aspirated mass of goat vitreous in grams

**Figure 18:**
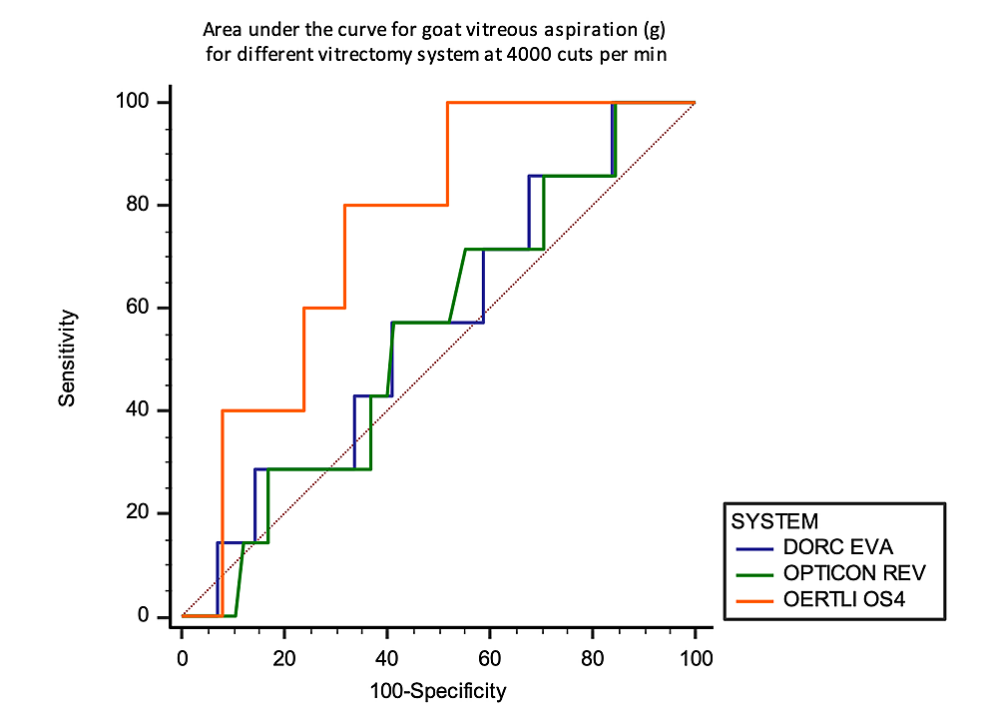
Area under the curve analysis at 4000 CPM against the aspirated mass of goat vitreous in grams

For goat vitreous, OS4 achieved a higher AUC of 0.752, 95% CI (0.548 to 0.956) at 4000 CMP (Figures 17 and 18).

## Discussion

To the best of our knowledge, this study is the first one of its nature in evaluating the efficiency of dual blade cutters of various standard vitrectomy platforms. Since the dual blade cutters have shown their superiority over single blade cutters in many studies, it may be safe to assume that most modern vitrectomies will be performed using these dual blade cutters in near future. Also, all the leading vitrectomy machine manufacturers have developed and are marketing their respective dual blade cutters due to proven better efficiency and fluidics. Although the concept of dual-port cutters is an old one only recently it has been applied in commercial use [13]. In a similar study where the standard single blade cutter was compared with the TDC cutter of EVA in an in vitro environment using a vitreous surrogate, it was noted that aspiration flow constantly decreased in the standard cutter when the cut-rate was increased to maximum capacity. This was contrary to the TDC cutter where the aspiration flow rate remained constant. These results were similar to the results of our experiment. Also, it was noted that for a given set of cut-rate and vacuum, the aspiration flow rate was higher for the TDC cutter as compared to the standard single blade cutter. These results were essentially replicated in vivo as well when the time required to perform core vitrectomy was taken as a measure of efficient vitrectomy while using the two cutters under identical parameters. The author compared 25- and 27-gauge TDC vitrectomes using 16,000 CPM with a single cut vitrectome. The author concluded that the mean duration to complete vitrectomy was significantly less (p<0.001) than when vitrectomy was performed with a single cut vitrectome [14]. This constant aspiration flow rate was similarly maintained across the three vitrectomy platforms when using goat vitreous in our study as well; the mass aspirated was more for machines with higher cut rates. This uniform aspiration flow rate was not maintained when albumin was used instead of goat vitreous as a vitreous substitute. We observed too frequent fluctuations in the egg albumin aspiration rate. This observation has also been made by Oravecz et al. in their experiment. In their study, they reported no significant increase or decrease in the mass aspirated (albumin) by dual blade cutters when compared to single blade cutters [7]. This may be due to the very different microstructure of egg white which is rich in albumin, globulins, and mucoproteins when compared to goat vitreous [15]. Although the experiment conducted by Oravecz et al. was conducted while using porcine vitreous whereas our experiment used goat vitreous, there was no significant difference in performance when using dual blade cutters, despite the fact that goat vitreous contains higher levels of hyaluronic acid and chondroitin sulfate as compared to human and porcine vitreous [16].

When comparing distilled water flow rate across three cutter platforms, we observed linearly increasing mass aspirated as the vacuum increased. Vitrectomy machine with the highest vacuum generation ability (revolution) had the most aspirated mass at the highest vacuum. There was no significant difference in mass aspirated as the cut-rate increased; also, the mass aspirated at a certain midrange identical setting also did not differ significantly across three platforms for this Newtonian fluid. This finding is similar to the experiment conducted by Oravecz et al. when comparing EVA and megaTRON S4 dual bladed cutters while using water as a vitreous substitute.

A similar experiment was conducted by Abulon and Buboltz where he compared Ultravit 23 and 25+ gauge cutters of Constellation Vision System (Alcon Laboratories, Inc., Fort Worth, TX, USA) in a model eye with water as a vitreous substitute. He showed that with port biased open, the flow rate decreased with an increasing cut-rate for any given vacuum setting. This observation is different from ours as we used dual blade cutters in all our experiments whereas a single blade cutter was used in Abulon’s experiment; thus showing the superiority of dual blade cutters at maintaining a stable flow rate across various machine settings [17,18].

Strength and limitations

The main strength of this study is the comparison of only dual blade cutters and uniform gauge. The machines were compared at constant values of CPM and vacuum and at their maximum potential. Three different vitreous surrogates were used. All data were obtained under standard conditions.

The main shortcoming is that we did not evaluate Constellation by Alcon because the dual blade cutter was not available in Pakistan. The other shortcoming is the use of goat vitreous instead of porcine vitreous (which is closer in its structure to human vitreous). Again, this could not be done due to a lack of ready availability.

## Conclusions

The higher cut rates and vacuum during vitrectomy enhance the efficiency of the machine when using dual blade cutters. Machines with inbuilt higher parameters perform more efficiently as compared to machines with lower parameters but this difference is maintained once we are using dual blade cutters only. This difference in performance is more evident when using water and goat vitreous as a human vitreous substitute. It is also to consider the importance of the results obtained through these two substitutes as they may, more precisely, mimic a real clinical situation encountered by retinal surgeons. This detailed data analysis also makes it easier for surgeons to chose the vitrectomy platform when considering efficiency in vitreous cutting as a paramount and decisive factor. This is to account for the very high procurement costs of these advanced vitrectomy machines in middle to lower-income countries.
